# Selective Mutism and Comorbidity with Specific Learning Disorders: Evaluation and Multimodal Intervention in a Clinical Case of a Female Child from 7 to 11 Years of Age

**DOI:** 10.3390/children11060746

**Published:** 2024-06-19

**Authors:** Micaela Capobianco, Alberto Costa

**Affiliations:** 1 Department of Economic, Psychological and Communication Sciences, Niccolò Cusano University, 00166 Rome, Italy; alberto.costa@unicusano.it; 2 IRCCS Fondazione Santa Lucia, 00179 Rome, Italy

**Keywords:** anxious-dependent attachment, internalizing problems, learning disorder, diagnostic comorbidity, cognitive–behavioral intervention, multimodal therapy

## Abstract

Selective mutism (SM) is an anxiety disorder that is characterized by a child’s persistent inability to communicate verbally in some or all contexts of social life. It is often associated with other cognitive–affective disorders. Performing cognitive–behavioral assessments and psychological interventions can be challenging due to the difficulty in administering standardized neuropsychological tests and involving family and teachers in the intervention program. In a single case study, a young Filipina girl with SM underwent a comprehensive neuropsychological assessment and received multimodal therapeutic intervention between the ages of 7 and 11. The psychological intervention included cognitive–behavioral psychotherapy to improve social–cognitive skills and learning abilities, reduce anxiety, and provide speech therapy. The parents and teachers were actively involved in the therapeutic process and a underwent a psycho-education program. Following this treatment, at the age of 11, the girl started verbalizing in therapy and school contexts, although she still used non-verbal strategies. There was also a gradual improvement in her communicative–linguistic skills and school learning. In conclusion, this report emphasizes the importance of applying an integrated and multimodal intervention to treat SM in children, including psychoeducation for parents and teachers.

## 1. Introduction

Selective mutism (SM) is an anxiety disorder (DSM 5, 2014) [1] that is characterized by a child’s persistent inability to communicate verbally in certain social contexts where verbal communication is expected. It is observed more frequently in girls than boys (2:1) (Capobianco, 2009; Capobianco, 2010) [2,3]. The notion of “refusal” has been replaced by “inability” to shift focus toward the cognitive processes and emotional states underlying SM (D’Ambrosio and Coletti, 2002 [4]; Vecchio and Kearney, 2005; 2006) [5,6]. SM usually arises between the ages of 3 and 6, but it is often diagnosed when the child starts school. The severity of the disorder varies, ranging from mild to severe. In some cases, the difficulty in verbal communication is specific to certain contexts or people, such as at school with peers or adults, and the child may resort to non-verbal strategies. In more severe cases, the child may struggle to communicate in almost any social context outside of the family, and even within the family, communication may be limited to just one parent, most frequently the mother (D’Ambrosio and Coletti, 2002 [4], Vecchio and Kearney, 2005; 2006) [5,6]. While children with SM typically have normal intelligence, the persistent presence of the disorder throughout preschool and school ages, particularly in school settings, could impact cognitive processes. Children with SM may experience difficulties in information processing and abstract reasoning, as well as reduced efficiency in executive processes, working memory, and language processes (Oerbeck and Kristensen, 2023) [7]. Moreover, comorbidities with specific learning disorders, attention deficit hyperactivity disorder (ADHD), and behavioral disorders have been documented (D’Ambrosio and Coletti, 2002 [4]; Steinhausen et al., 2006 [8]; Sloan, 2007 [9]; Yeganeh et al., 2003; 2006) [10,11]. It is hypothesized that the absence of language use and reduced social participation over time may deplete basic cognitive processes, such as working memory, attention, and learning acquisition processes. These factors could contribute to the maintenance and exacerbation of SM, potentially increasing the child’s sense of inadequacy and low self-efficacy (Mayworm et al., 2015) [12].

When it comes to understanding the cognitive and emotional mechanisms behind the behavior of individuals with selective mutism (SM), evidence from scientific studies and clinical observations highlights the following aspects (Capobianco and Cerniglia, 2018; [13], Driessen et al., 2020) [14]: (1) Feelings of inadequacy and inability: Individuals experience discomfort in new situations, feel incapable, and devalue themselves; (2) Fear of judgment by others: They believe that others will negatively judge their actions; (3) Feelings of shame and meta-shame: They fear experiencing shame and worry that others will perceive this feeling. The above aspects are related to the following cognitive biases: (1) Catastrophizing the consequences of their own mistakes; (2) Overgeneralizing: They fear and perceive inability in any unfamiliar situation; (3) Selective abstraction concerning their inability; (4) Underestimation of their resources. As a result, individuals with SM are susceptible to signals, attention, and the looks of strangers. They may also display hypersensitivity to criticism.

Regarding the development of selective mutism (SM) in preschool and school-age children, there is a wide range of outcomes. For some individuals, the disorder tends to persist chronically, while a gradual decrease in symptoms is observed in others. However, even in cases of improvement, noticeable difficulties in speaking may persist throughout childhood and into the school years. The prognosis is influenced by various factors, including early diagnosis and tailored intervention, the presence of other disorders, family support, and collaboration across different contexts of the child’s life (Mercado, 2024) [15]. Due to the complex cognitive and behavioral profile often seen in individuals with SM, it is crucial to implement a comprehensive intervention approach that is consistent across various aspects of the child’s life (Bergam et al., 2012 [16]; Steinhausen et al., 2006 [8]). Specifically, the cognitive–behavioral approach should focus on enhancing communication skills, reducing anxiety, improving nonverbal strategies, and preventing the tendency to generalize and catastrophize. It is essential for families and teachers to understand the importance of their involvement in the intervention program and to be familiar with the specific characteristics of the disorder. (Capobianco and Cerniglia, 2018) [13].

## 2. Study Objectives

To the best of our knowledge, there have been few long-term studies that have aimed to investigate the effectiveness of a comprehensive intervention model on the progression of selective mutism based on the unique neuropsychological profile of each individual. Some factors, such as the challenges in conducting personalized language-related tests, the presence of other health conditions, and the difficulty in maintaining active involvement of family and teachers in the intervention program, may have posed significant constraints.

This study aims to describe the neuropsychological assessment and intervention process used for a female Filipino child with selective mutism (SM) from the first grade (age 7) to the fifth grade (age 11). A thorough neuropsychological assessment was conducted before the start of the intervention at age 7 (T0) and after the completion of the intervention at age 11 (T1). We will report and discuss the specific characteristics of the integrated intervention model we implemented as well as the results.

## 3. Methodology

### 3.1. Participant

The neuropsychological assessment and intervention process involved a female child of Filipino origin (referred to as G and “child” as G, on following pages) born in Italy on 5 May 2011. She was in grade I of elementary school and had been diagnosed with selective mutism (SM) alongside mixed developmental disorder (challenges in language comprehension and expression) [ICD-10, F83] of a moderate degree. These conditions were identified during her preschool years at the Health Reference Company in Italy, where her family resided. The Health Reference Company, in collaboration with a health association called Bimbo al Centro ETS, was seeking a comprehensive psychometric and clinical evaluation by a multidisciplinary team. This evaluation aimed to guide an integrated and targeted intervention path, employing a cognitive–behavioral approach along with speech therapy rehabilitation focused primarily on improving receptive language skills. When gathering the medical history, both parents, who have limited proficiency in Italian, were interviewed with the assistance of an Italian family friend acting as a language mediator. According to the parents, they had always had a reserved nature, finding it challenging to communicate with unfamiliar individuals, and they tended to be less involved in social activities outside the family. These difficulties were further compounded by cultural differences.

### 3.2. Anamnestic Data

The parents reported that the baby was born at full term (39.3 weeks), weighing 3000 g, with a weight appropriate for gestational age (AGA). They primarily spoke Italian with the baby, even though it might not have been grammatically correct Italian. The baby was indirectly exposed to the Filipino language through listening and observing family interactions. The parents reported that the baby’s language development was somewhat slow, with verbal production inconsistent with their age. The child had always been very inhibited and shy around unfamiliar people and had few social relationships beyond school. Due to work commitments, the child had not attended classmates’ birthday parties or other school events. The child spoke only at home with her family and seemed to have significant difficulties in acquiring school learning.

In unfamiliar situations, the child exhibited selective mutism, which was not compensated for by nonverbal communication such as gestures, facial expressions, lip-reading, or drawing. The child also avoided eye contact. The teachers were not familiar with selective mutism and did not know how to help the child, who showed significant isolation and lacked alternative means of communication with adults and peers.

The teachers had been trying to encourage verbal expression by making explicit requests and asking questions, even when other students were present. However, this approach seemed to be ineffective, considering the emotional and cognitive characteristics associated with mutacic behavior (Capobianco and Cerniglia, 2018) [13].

It had been decided, with the agreement and consent of the parents, to conduct a more comprehensive initial assessment to gain a better understanding of G’s overall cognitive functioning, neuropsychological abilities, and most importantly, the specific thought processes and emotions underlying G’s selective mutism. This understanding is crucial for implementing targeted cognitive–behavioral psychotherapy (D’Ambrosio and Coletti, 2002; Capobianco and Cerniglia, 2018) [4,13]. There was a plan to conduct parent training sessions and interviews with the child’s teachers to familiarize them with the characteristics of selective mutism and to discuss relational and behavioral strategies (in line with the Ministry of Health Guidelines) to be employed collaboratively with the child at home and at school. Providing appropriate psychoeducation to adults who spend a significant amount of time interacting with the child is a vital component of the rehabilitation program. (Capobianco Cerniglia, 2018; Catchpole et al., 2019) [13,17].

The evaluation was conducted in two stages: an initial assessment at the commencement of intake, when the individual was 7 years old and in the first year of primary education (T0), and a subsequent assessment during the individual’s fifth year of primary education at the age of 11 (T1), following a sustained regimen of rehabilitation, as detailed in the Multimodal Intervention Program referenced in this research.

### 3.3. The Assessment (7 Years-End I Primary): T0

#### 3.3.1. Relational–Behavioral Aspects

G. showed interest in the proposed activities upon entering the room. The specialist aimed to build a therapeutic alliance and establish a trusting relationship with G. by not insisting on verbal communication. Instead, they encouraged nonverbal communication through a neutral attitude, creating an accepting environment. The specialist applied a behavioral strategy based on symptom extinction, allowing the child to answer questions using gestures, drawing, and pointing to communicate. G. preferred drawing and was quite skilled at it. The specialist was able to administer tests without the need for verbal communication. G. responded to questions with gestures, head nods, and drawings. Despite an elusive gaze, G. often looked at the operator to express her emotional state during tasks.

#### 3.3.2. General Cognitive Functioning and Underlying Processes

The child demonstrated above-average nonverbal intelligence, as indicated by the scores on Raven’s Progressive Matrices (CPM) scales, which corresponded to the 90th percentile. While the complete administration of the WISC-IV cognitive scales was not feasible, the nonverbal subtests comprising the visuo-perceptual reasoning (RP) and processing speed (VE) index were administered, and the weighted scores (WS) for each task are provided below.

The Table 1 shows G.’s cognitive non-verbal skills assessed with WISC-IV cognitive scale (WISC-IV: Non-verbal Evidence-STEP T0).

As observed in Table 1, the cognitive assessment regarding the visuo-perceptive reasoning (VPR) and processing speed (SP) index confirm the results that emerged from the Raven’s progressive matrices (MPR) scales concerning the good level of reasoning mediated by visuo-perceptive skills. This allowed us to add specific information to other underlying cognitive processes that were found to be deficient. The child showed significant difficulties in “pencil paper” activities that required reasoning mediated by the executive speed of the graphic act (cipher and symbol search), then in activities based on good functioning on the visuomotor plane for fluency, accuracy, and speed. The child’s processing speed was found to be deficient. Difficulties were also observed in the skills of reproducing a two-dimensional patterns through cubes (Drawing with cubes, PP 5*, below average) or in pencil copying of increasingly complex geometric figures in the pattern copying subtest of the Perceptual and Visuomotor Integration Test (IVP), in which the child scored between the 10th and 15th percentiles. The ergonomic aspects were borderline, including non-dynamic pen prehension and ill-defined strokes with discontinuous pressure. Thus, at 7 years, difficulties were evident in the visuomotor, visuoconstructive, and executive speed levels in the face of a good level of reasoning mediated by visuoperceptive skills.

#### 3.3.3. Communicative–Linguistic Skills

Expressive, discursive, and narrative skills were assessed using the spontaneous language analysis methodology of a video recording of the child’s spontaneous production in the family context. The child exhibited a morphosyntactic level inconsistent with chronological age, producing complete but not complex nuclear sentences with significant phonological and morphological alterations that sometimes impacted the overall intelligibility of verbal production. The child’s discursive and narrative skills were also not age-appropriate. Linguistic prosody and turn-taking were not functional for effective communication. Verbal comprehension, assessed by the Morphosyntactic Comprehension Test (PVCM) (Rustioni and Lancaster, 2007) [18], indicated that the child’s performance was not age-appropriate, scoring in the 57.5th percentile, which is classified as “low–medium” for a 7-year-old. The child understood situations related to contingent reality but had difficulties with metalinguistic tasks and comprehending decontextualized sentences that lacked a concrete counterpart.

#### 3.3.4. Learning Abilities

Reading, writing, and other aspects of school learning could not be fully assessed in the clinical setting at this early stage of neuropsychological evaluation. Therefore, key information about the child’s learning acquisition processes was gathered through teacher interviews and an analysis of her school notebook work. The teachers reported that the child’s progress in learning was not in line with age and educational exposure, regardless of her mutacic behavior. The basic grapheme–phoneme process for reading and writing in print was not yet automated. Additionally, the child had difficulties in the logical–mathematical area, including tasks such as number dictation, simple operations, enumeration, and mental calculation. Despite these challenges, the teachers noted that the child showed good attention in class and general cooperation during tasks, although they observed a general slowness in task execution. At 7 years old, the child was at high risk of developing a specific learning disorder.

#### 3.3.5. Exploration of Thoughts–Emotions–Behaviors (ABC in the Context of Cognitive–Behavioral Therapy)

From the projective test “The Family Drawing”, as well as clinical observation of behavior and exploration of thoughts and emotional states (ABC—event–thoughts–emotions–behavior) (Vecchio and Kearney, 2006) [6], elements of inhibition, social anxiety, shame and meta-shame, self-perception as inadequate, insecurity, and self-evaluation emerged. Specifically, significant aspects of social–emotional distress related to traits of isolation, infantilism, and dependence on the family group were revealed through “The Family Drawing”. A key therapeutic goal was to explore the prevailing emotions, behaviors (C), and dysfunctional thoughts (B) underlying G’s fear of talking (ABC analysis), which was primarily achieved through the following.

Playful simulations with puppets or dolls and drawing invented comics were used to represent various daily events where the fear of speaking could arise and intensify (e.g., responding to a teacher’s questions at school in front of classmates, encountering an unfamiliar adult in the street with their mother). The analysis of thoughts and emotions revealed that G. experienced significant difficulty and a perceived an “inability” to speak, especially at school when she was asked to perform or becomes the center of attention. These situations were perceived as dangerous to her sense of effectiveness and self-image. G. particularly feared judgment from others, was afraid of making mistakes, being laughed at, or made fun of (“everyone will laugh”), and felt ashamed while fearing that others would notice her shame (meta-shame). Depressive themes of catastrophizing about the consequences of her speech on others prevailed in G., and the fear of being teased was closely linked to a perception of inadequacy and low self-evaluation (“I’m afraid of making a mistake”). Her language delay and awareness that she did not fully understand the verbal stimuli around her and produced incorrect speech heightened her anxiety, thoughts of inadequacy, and inhibition, thus maintaining her mutacic behavior. Additionally, factors such as family isolation contributed to maintaining elements that stabilize and chronicle the cognitive biases automatically activated in dreaded unfamiliar situations. Poor use of expressive skills and difficulties in language comprehension further hindered the improvement of abstraction and intention-expression skills, increasing her condition of isolation and fear of making mistakes. According to the scientific literature, the odds of acquiring learning skills (reading, writing, and logical–mathematical areas) are lower among children with a prior or current language disorder, with a stronger correlation if the specific language disorder is of a mixed type. (Capobianco, 2010; Ponzurick, 2012) [3,19].

## 4. Multimodal Intervention Program

According to the guidelines for intervention for children with SM, a multidisciplinary and integrated approach based on cognitive–behavioral strategies was applied, involving the family and school context. The main objective was to share interaction strategies and enhance awareness of the clinical, cognitive, and emotional characteristics of G’s disorder. The individual rehabilitation program included a course of cognitive–behavioral psychotherapy, followed by speech therapy to address receptive aspects of language and enhance lexical and morphosyntactic comprehension. During speech therapy, the specialist worked with the child to improve her practice–executive skills, enhancing visuomotor and visuoconstructive abilities. After approximately two years of individual psychotherapy, conducted weekly for about an hour each session, the child was placed in a workshop with a peer. This setting aimed to address cognitive and emotional aspects within the context of peer interaction. Laboratory meetings with another child were held weekly for about 1.5 h. During these sessions, consistent nonverbal communication strategies were used, including pictures, spontaneous drawings, and written words (even single words).

The workshop is still ongoing. The child has attended the clinical center consistently, with few absences.

The main and basic aims of psychotherapy have been: (1) to decrease the level of generalized anxiety; (2) to modify rigid and automatic thoughts by operating a cognitive restructuring of the interpretation of daily life situations in which mutacic symptomatology occurs, playfully proposing alternative strategies with different resolutions.

Below are some cognitive–behavioral strategies used with the child based on processes of acceptance and reflection on alternative interpretations of events.

1. Decrease anxiety and modify dysfunctional thoughts of:

1. Catastrophizing and overgeneralizing about speaking and judgment from others: Speaking will not necessarily result in “negative judgments” from others. Therefore, reflect on alternative assumptions about others’ judgments and recognize that speaking can lead to different outcomes and is often more convenient and useful than not speaking. Also, understand that it is normal for some people not to like us, but this does not harm our value and abilities. Reflect on shame as a natural emotion that may occur at certain times. Work on the basic belief, “We are not ashamed, nor should we show shame”, which is often associated with a sense of diminished self-worth and the compromised goal of always maintaining a good image. 2. Self-worth and perceived inability to speak: Promote autonomy and self-confidence. Understand that others do not always laugh at what we say, and if someone disagrees with our speech, it does not mean we lack value. Focus on accepting others’ perspectives and reducing the cause–effect relationship between their judgment and our self-worth.

2. Promoting and prompting attribution, expression, description, and reflection of emotions:

Through simulated events such as games and drawings, G. was repeatedly asked to indicate the emotion she thought the character was feeling at that moment. This was done either by pointing to faces, each representing a specific emotion, or by writing. This approach aimed to promote reflection on the relationship between events, emotions, and their consequences.

During therapy sessions, the child was exposed to alternative thoughts, emotions, actions, and consequences of the events simulated in the game and/or drawing, contrasting them with those described by G. This was achieved through acting out the simulated events and/or engaging in written dialogue. The aim was to encourage reflection on several key points: 1. the value of speaking and the costs/benefits of silence: reflecting on the usefulness of speaking and considering the potential advantages and disadvantages of remaining silent. 2. Acceptance of disagreement: Acknowledging that it is normal for someone not to “like” what we say, emphasizing the independence of our self-value from the negative or positive evaluations of contingent situations (e.g., being questioned, speaking in front of strangers, etc.). 3. Acceptance of shame: recognizing shame as a natural emotion that everyone experiences and emphasizing that it does not necessarily have an exclusively negative meaning.

Below is an example of simulated situations with invented characters used in therapy: simulations with the child of alternative hypotheses about how the story might end concerning different characters (thoughts, actions, emotions).

In a simulated scenario featuring puppets, a typical interaction unfolds: a mother and her child encounter a friend of the mother (an adult) with her own child. During this encounter, the friend’s child invites G. to come over to their house one afternoon to play. This prompts an exploration of G’s thoughts, emotions, and subsequent behaviors in response to this initial event, alongside an examination of the consequences of these actions. Following this initial exploration, the therapy session progresses to the next phase, where alternative thoughts, emotions, and behaviors are proposed and discussed with the child. This process encourages G. to consider different possible outcomes resulting from alternative responses. By presenting diverse perspectives stemming from the same scenario, the therapy session aims to foster an understanding of the varying consequences associated with different actions. For example, one potential outcome may involve G. accepting the invitation and going over to the friend’s house to play, resulting in both children feeling joyful about spending time together.

To facilitate effective communication for the child, various colorful pictures within augmentative and alternative communication (AAC), including the picture exchange communication system (PECS), were utilized. These images could depict simple responses like YES and NO, emotions, or basic sequences of actions. The paper-based images were complemented with short videos on the computer or drawings, providing the child with multiple nonverbal communication options and diverse methods for responding to questions. Table 2 illustrates an example event (A) alongside two alternative thoughts (B), both of which may lead to positive outcomes in resolving the interaction.

### 4.1. The Main Aims of the Interaction in the Family Setting

It was essential to conduct psychoeducational meetings to address the issue of selective mutism. These sessions aimed to clarify that G’s silence was not due to refusal, tantrums, or oppositional behavior, but rather because she experienced a discomfort that rendered her unable to speak. This inability to verbalize was rooted in significant anxiety. The primary purpose of these meetings was to alter the parents’ understanding of G’s behaviors, thereby reducing the burden of responsibility placed on the child.

The following general suggestions on how to interact with G. were provided to her parents:

1. Adopt a neutral attitude: Maintain a neutral stance regarding G’s silence. Avoid frequently highlighting it as a problem or displaying a punitive attitude. 2. Encourage independence in daily activities: Do not substitute for G. in her daily activities and interactions. When someone asks her a question, allow her the space to respond without immediately insisting on a verbal answer or answering for her. Encourage her participation in conversations by accepting alternative modes of communication. Similarly, do not complete her homework for her or anticipate her answers; instead, assist her in thinking more critically and becoming more aware of her own study strategies. 3. Promote autonomy at home: Gradually encourage G. to take on small daily tasks to build her independence, such as paying the newsagent, making a phone call, or asking for information. 4. Increase social interactions: Facilitate more social gatherings with G’s peers outside the home by organizing class events, accepting invitations to other children’s homes, or inviting classmates and friends over.

### 4.2. The Main Aims of the Interaction in the School Setting

It was crucial to maintain a consistent approach to behavior with G. both at home and school to maximize the effectiveness of her intervention. Psycho-education for the teachers included adopting a neutral attitude towards G’s silence, reinforcing nonverbal communication with teachers and peers, and utilizing alternative materials such as drawing, writing, multiple-choice questions, computers, and interactive whiteboards (IWB). Additionally, the teachers should promote and create small group activities with at least one peer G. feels comfortable speaking with, avoid situations that might embarrass or shame her, and acknowledge communication efforts, whether nonverbal or verbal, without explicitly emphasizing verbal communication. For learning acquisition difficulties, various compensatory and dispensatory strategies tailored to her needs, such as concept maps, captions with pictures, and voice readers, were gradually implemented.

## 5. Follow-Up Assessment and Results of Intervention (11 Years Old vs. Primary: Step T0)

At the end of the fifth grade, a new assessment was conducted, revealing the following profile in specific domains mediated by nonverbal skills: visual–perceptual reasoning (RPV) scored 108 (60th percentile). In contrast, processing speed (SP) scored 75 (10th percentile, below average). The individual subtests had the following weighted scores (SW): drawing with cubes: 11; reasoning with matrices: 14; illustrated concepts: 9; cipher: 4*; symbol search: 8*. The results confirmed a good level of general cognitive functioning, with a slight improvement in processing speed, although it remained below average, compared to good functioning in visuo-perceptive channel-mediated reasoning. Following the speech rehabilitation course (as described in the first intervention cycle), there was evidence of improvement in visuo-constructive skills, although a fragility in processing speed persisted, particularly in pencil–paper activities involving executive functions. Overall, all the tests confirmed aspects of executive slowness and fatigability, which increased with the duration of the activities.

After the rehabilitation cycle, G’s participation in various relational dynamics significantly improved through the spontaneous use of nonverbal communication strategies, particularly writing on paper or a blackboard to express her thoughts and emotions. A more spontaneous use of nonverbal communication was generally observed, especially toward teachers and adults. This increase in spontaneity and communicative behavior was likely related to a significant reduction in generalized and selective anxiety and a decrease in cognitive biases related to the fear of others’ judgments.

The teachers report that although G. was still slow in processing, she had become more autonomous and now communicated her most important needs more spontaneously using gestures, facial expressions, and writing. She actively sought peer interaction more frequently and had begun to respond verbally to certain teachers during activities conducted outside the classroom. Thus, the use of verbal communication had emerged in the school context, albeit exclusively with some teachers outside the classroom.

Regarding learning skills, multiple tests were administered without the assistance of family members, and the child responded using nonverbal methods, writing, and occasionally with short verbal sentences. This enabled a thorough assessment of the writing skills acquired between the ages of 7 and 11, following the rehabilitation cycle, with occasional verbal input with a lower voice.

The Battery for the Assessment of Writing and Orthographic Proficiency in Compulsory Schooling-3 (BVSCO-3) (Tressoldi andCornoldi, 2012) [20] was administered, consisting of tests on dictation, narration, and writing speed. The child exhibited a mixed number of errors, typically indicating a “Demand for Attention”. She demonstrated poor fluency and slowness in cursive writing. Below are the results summarized in Table 3.

### 5.1. Reading

The child agreed to read aloud for the specialist, during which MT reading tests were administered (Cornoldi, and Colpo (2004)) [21]. The results at the end of the 4th grade showed below-average correctness (request for immediate intervention) and speed (request for attention) in reading, with aspects of intonation and punctuation falling short of standard expectations. In narrative text comprehension (informational passage at the end of 4th grade), the child answered 4 out of 10 questions, indicating a need for attention. These findings suggest difficulty in reading comprehension.

### 5.2. Communicative–Linguistic Area

The BVL 4–12 Battery for Language Assessment in children aged 4 to 12 years (Marini, Marotta, Bulgheroni, Fabbro, 2014) [22]. was utilized, with only the verbal comprehension test administered. Improvement was observed in the area of verbal comprehension, with the score now within normal limits for the child’s chronological age, indicating progress from the deficit level noted in the initial assessment.

Additionally, an analysis of expressive and narrative skills was conducted in this second step, utilizing spontaneous production collected at home via video recording.

The analysis of spontaneous production indicated that the child’s morphosyntactic level remained below her chronological age, despite a notable improvement in discursive and narrative skills compared to the initial assessment at the end of elementary school. While her spontaneous speech, assessed through video recordings at home, still lacked grammatical richness and showed incomplete mastery of morphological and syntactic structures in Italian, there was a marked enhancement in expressive skills since the first assessment. Furthermore, while lexical and grammatical comprehension skills improved, they were not yet fully aligned with the child’s chronological age. The scores obtained from the BVL battery suggest a borderline level, although not as deficient as in the initial assessment.

### 5.3. Logical–Mathematical Area

In the second phase of assessment, we were able to administer a standardized test to evaluate the child’s mathematical skills. In contrast, during the first phase, the child’s acquisition processes were reported by teachers at the end of first grade. The AC-MT-3 test (Cornoldi, Mammarella, Caviola, 2020) [23] was administered to assess her calculation and mathematical reasoning skills.

During the second evaluation, an updated diagnosis revealed a comorbidity with a mixed-type specific learning disorder (SLD). The child demonstrated considerable difficulties in the logical–mathematical domain, particularly with written operation procedures, number transcoding, judging numerosity, and sorting numbers. 

## 6. Summary of Results

This study examined the longitudinal assessment of a girl diagnosed with selective mutism and comorbid specific language disorder (SLD) from ages 7 to 11. Assessments were conducted before (T0) and after (T1) a comprehensive, multimodal intervention. After the initial neuropsychological evaluation (T0), the child underwent an integrated treatment program, including speech therapy to enhance communicative–linguistic and visuomotor skills, cognitive–behavioral psychotherapy (both individually and with a peer), parent training, and psychoeducation for teachers on effective strategies. The intervention was consistently provided weekly to the child and monthly to the school.

From the initial assessment at around age 7, before the longitudinal cycle of integrated intervention, the child exhibited a significant expressive and receptive language disorder, along with notable deficits in visuomotor and visuoconstructive skills and processing speed. The mutacic behavior was highly pervasive, and the child did not employ any alternative communication strategies, rendering further standardized testing for school learning infeasible, even through nonverbal communication. An assessment of her underlying thoughts and emotions revealed a high level of generalized anxiety, fear of being evaluated as inadequate, and intense shame. The child’s parents led a largely isolated social life, and the teachers were unaware of effective relational strategies, as recommended in the scientific literature, for working with children with MS.

Psychotherapeutic work focused on cognitive restructuring of dysfunctional thoughts, and reducing generalized anxiety and shame was essential. This approach gradually led to a decrease in the child’s social anxiety and increased her participation in interactions through nonverbal communication strategies accepted and shared at school. By age 11 (T1), in the fifth grade, it became possible to conduct a comprehensive neuropsychological assessment of her school learning, leading to a clearer and more complete clinical diagnosis. This revealed that her selective mutism was associated with a mixed learning disorder, with particular difficulties in the logical–mathematical area and reading (specifically in decoding rather than text comprehension). In the fifth grade, the child began speaking to some teachers and responding to oral questions, although this was done outside the classroom. During the second assessment (T1), post-treatment, she showed improvements in communicative–linguistic skills, particularly receptive skills, and a significant decrease in anxiety, shame, and fear of judgment. This allowed for increased participation in social interactions through nonverbal communicative strategies with peers and unfamiliar adults. The reduction in mutacic behavior, along with changes in underlying thoughts and emotions, enabled the administration of standardized tests to more comprehensively assess her learning and cognitive processes. Table 4 summarizes the changes in the child’s cognitive abilities, learning skills, and mutacic behavior from T0 to T1. Skills that did not show improvement over time are marked with an asterisk (*).

As depicted in Table 4, following the integrated treatment, the child’s non-verbal cognitive abilities remained consistent, while challenges with visuomotor skills and executive slowness persisted. Notably, there was an improvement in visuo-constructive skills.

With amelioration in selective mutism symptoms and language proficiency, the second assessment (T1) facilitated a comprehensive evaluation of academic progress, crucial for further intervention.

By the age of 11, the child had attained a level of communication, primarily non-verbal, and social engagement adequate for collaborative learning endeavors with peers and educators in school.

## 7. Critical Discussions and Application Conclusions

Clinical and research findings indicate a strong resemblance between selective mutism (SM) and social anxiety or social phobia, with SM often considered to be a subtype of anxiety spectrum disorders (DSM 5, 2014; Cunningham et al., 2006; Yeganeh et al., 2003; 2006) [1,10,11,24]. The case of G. corroborates this notion and underscores the multifaceted nature of SM, influenced by biological, psychological, and environmental factors across various life contexts. In this light, parental and teacher involvement is crucial to align intervention goals with the child’s needs across different settings. This report underscores the significance of early diagnosis and thorough neuropsychological assessment, essential for understanding the cognitive and emotional underpinnings of SM and identifying comorbidities or specific challenges that may perpetuate the disorder. For example, G. initially presented with a mixed-type language disorder, later followed by the detection of a specific learning disorder, offering insights into the extent of her SM. However, challenges in administering standardized language-mediated assessments should be considered, prompting the utilization of non-verbal tests and involving family members and teachers in analyzing spontaneous behaviors.

The primary aim of the intervention program for G. centered on reducing generalized anxiety. Through simulated events in games and drawings, the program focused on reflecting and restructuring biases underlying selective mutism (SM) across several key areas: (i) understanding the utility of speaking and weighing the costs and benefits of remaining silent; (ii) embracing acceptance of others’ judgments while disentangling self-worth from external evaluations in various situations, such as public speaking or interacting with teachers; (iii) recognizing and embracing emotions like fear and shame. G’s typical response to unfamiliar situations involved avoidance and withdrawal, likely as a defense mechanism against perceived distress. Notably, a dependent attachment style, family social isolation, and parental behaviors often exacerbated G’s anxiety and reinforced inhibitory behaviors (Allison et al., 2023) [25]. Thus, the psychoeducational program aimed to educate both parents and teachers about the phenotypic expression of SM and implement more effective, collaborative, relational, and communicative strategies. Initially, her parents and teachers believed that G. voluntarily chose not to speak, prompting continuous encouragement for verbalization. However, discussions highlighted evidence that G. was genuinely unable to speak in certain circumstances due to underlying emotional and cognitive processes. Increased awareness among her parents and teachers regarding the unintentional nature of G’s behaviors led to modifications in their responses and interactions with G. in daily life (Shorer et al., 2023; Wang and Monga, 2023, Koskela et al., 2023) [26,27,28]. Over time, G. began verbalizing in front of new teachers and peers, particularly during the transition from primary to secondary school, suggesting a gradual reduction in anxiety related to speaking difficulties. Changes in classmates and teachers may have also contributed to this shift. In elementary school, children with SM often face labeling and prejudice, reinforcing self-perceptions of shyness and fragility. However, cognitive restructuring aimed at reducing anxiety levels and altering absolutist self-perceptions allowed for the construction of a new self-representation and improved self-efficacy (Capobianco, 2009) [3].

## Figures and Tables

**Table 1:** WISC-IV (nonverbal evidence)—STEP T0—age 7 years: Only tests in which G scored below or above the standard score average (WS) are marked with asterisks (*).

Visuo-Perceptual Reasoning (VPR)	Weighted Scores (WS)	INDEX (VPR)
*Reasoning with Matrices*	14	Above average
*Drawing with cubes*	5 *	Below average
*Illustrated concepts*	12	Above average
TOT. QI RV	31	QI 102
Processing Speed (SP)	WS	
*Cipher*	5 *	Below average
*Search for symbols*	6 *	Below average
TOT. QI SP	11	QI 74 *

**Table 2:** Alternative thoughts (B) of an event (A).

**A** **(Event)**	
The child asks the little girl if she wants to come and play with him at her house	
**B (alternative thoughts) 1**	**B (alternative thoughts) 2**
*“What if you don’t like me?”*	“*That’s great, I’d like to go and play at his house. We’re going to have a blast.*”
*“It doesn’t matter, I’ll try…” “I can’t be nice to everyone. I’ll try anyway.”*	

**Table 3:** Writing tests: handwriting, dysorthography, writing speed (T1). Skills with “*” are below average (Request for Attention).

Track Dictation *	
	Phonological errors: 5 −0.95 ds REQUEST FOR ATTENTION
	Non-phonological errors: 4 −0.87 ds ATTENTION DEMAND
	Doubles/accents: 3 −0.90 ds REQUEST FOR ATTENTION
	errors: 16 −0.60 ds 15° REQUEST FOR ATTENTION
Production of Written Test—Narration *	
	Words: 33 −0.70 ds 15° ATTENTION DEMAND
	Errors: 6 Percent total error: 18% −2.66 ds <5° REQUEST FOR IMMEDIATE ACTION
Speed of Writing *	
“le”	Graphemes: 45 −0.30 ds 15° ATTENTION DEMAND

**Table 4:** Initial abilities (T0) and post-treatment abilities (T1). Skills that did not show improvement over time are marked with an asterisk (*).

Cognitive Abilities Non Verbal QI: 108	T0 (First of Multimodal Program)	T1 (Post Multimodal Program)
Visuomotor skills *	Below average	Below average
Visuo-constructive skills	Below average	Slight improvement
processing speed *	Below average	Below average
LEARNING ABLITIES	Not valuable with tests	It was possible to administer tests and delineate neuropsychological functioning
LANGUAGE		
Comprehension skills	Severe disorder	Slight improvement
Verbal skills	Severe disorder	Slight improvement
MUTISM	Severe with social anxiety and no mode of communication with teachers and peers at school	Moderate: minor anxiety and use of non-verbal communication, sometimes verbal. More participation in shared activities with peers. The child talks to some teachers and answers oral questions

## Data Availability

No new data were created or analyzed in this study. Data sharing is not applicable to this article due to privacy restrictions.
